# Can Air-Conditioning Systems Contribute to the Spread of SARS/MERS/COVID-19 Infection? Insights from a Rapid Review of the Literature

**DOI:** 10.3390/ijerph17176052

**Published:** 2020-08-20

**Authors:** Francesco Chirico, Angelo Sacco, Nicola Luigi Bragazzi, Nicola Magnavita

**Affiliations:** 1 Post-Graduate School of Occupational Health, Università Cattolica del Sacro Cuore, 00168 Roma, Italy; nicola.magnavita@unicatt.it; 2 Health Service Department, State Police, Ministry of Interior, 20125 Milan, Italy; 3 Local Healthcare Unit Roma 2, 00155 Roma, Italy; angelo.sacco@alice.it; 4 Laboratory for Industrial and Applied Mathematics (LIAM), Department of Mathematics and Statistics, York University, Toronto, ON M3J 1P3, Canada; bragazzi@yorku.ca; 5 Department of Woman/Child & Public Health, Fondazione Policlinico A. Gemelli IRCCS, 00168 Roma, Italy

**Keywords:** outbreak, airborne transmission, SARS-CoV-1, MERS-CoV, SARS-CoV-2, ventilation, prevention, safety, workplace

## Abstract

The airborne transmission of SARS-CoV-2 is still debated. The aim of this rapid review is to evaluate the COVID-19 risk associated with the presence of air-conditioning systems. Original studies (both observational and experimental researches) written in English and with no limit on time, on the airborne transmission of SARS-CoV, MERS-CoV, and SARS-CoV-2 coronaviruses that were associated with outbreaks, were included. Searches were made on PubMed/MEDLINE, PubMed Central (PMC), Google Scholar databases, and medRxiv. A snowball strategy was adopted to extend the search. Fourteen studies reporting outbreaks of coronavirus infection associated with the air-conditioning systems were included. All studies were carried out in the Far East. In six out the seven studies on SARS, the role of Heating, Ventilation, and Air Conditioning (HVAC) in the outbreak was indirectly proven by the spatial and temporal pattern of cases, or by airflow-dynamics models. In one report on MERS, the contamination of HVAC by viral particles was demonstrated. In four out of the six studies on SARS-CoV-2, the diffusion of viral particles through HVAC was suspected or supported by computer simulation. In conclusion, there is sufficient evidence of the airborne transmission of coronaviruses in previous Asian outbreaks, and this has been taken into account in the guidelines released by organizations and international agencies for controlling the spread of SARS-CoV-2 in indoor environments. However, the technological differences in HVAC systems prevent the generalization of the results on a worldwide basis. The few COVID-19 investigations available do not provide sufficient evidence that the SARS-CoV-2 virus can be transmitted by HVAC systems.

## 1. Introduction

Many countries have responded to the “Coronavirus disease 2019” (COVID-19) pandemic caused by the emerging infectious agent termed as “Severe Acute Respiratory Coronavirus type 2” (SARS-CoV-2) by adopting stringent containment measures, such as the so-called lockdown. While these behavioral, non-pharmacological interventions have been proven effective in curbing the spread of the virus [1,2], they are having, on the other hand, significant economic and social consequences [3,4] and cannot, by their nature, be implemented and enforced indefinitely [5]. Several governments have started gradually easing and relaxing restrictions, by reopening selected businesses and production activities. The resumption of daily activities is now taking place in some countries, during the summer season, when the use of air-conditioning systems in indoor environments is necessary to ensure comfortable working conditions. The question therefore arises whether the use of Heating, Ventilation, and Air Conditioning (HVAC) systems can contribute to the further spread of the microorganisms in the workplace. Being a novel virus, the precise mode and routes of transmission of SARS-CoV-2 are still debated.

SARS-CoV-2 is thought to primarily spread from human to human, by close contact transmission, as well as through respiratory droplets of >5–10 μm in diameter, which are generated when an infected person coughs, sneezes, talks, or sings at short distances, generally <1–2 m [6,7]. The “airborne” transmission of droplet nuclei (“aerosolized particles of <5 μm in diameter”), remaining infectious when suspended in the air over long distances and time, is still controversial [7]. Indeed, it was found that some individuals behave as “speech super-emitters,” releasing SARS-CoV-2 particles of ≤1 µm in diameter during normal breathing and speech [8], coughing and talking [9,10]. Some airborne “superspreading” events have occurred in the past with other coronaviruses, e.g., in 2003, a SARS-CoV-1 outbreak initiated in Hong Kong and spread to other countries, and in 2012, a “Middle Eastern Respiratory Syndrome” (MERS)-CoV outbreak in the Kingdom of Saudi Arabia and in 2015 in South Korea [11,12,13]. Moreover, the airborne transmission of SARS-CoV-2 can occur during medical procedures that generate aerosols (“aerosol-generating procedures”) [14]. Many aerosol-generating surgical procedures have been identified [15,16,17,18,19,20], and special preventive measures have been proposed [21,22]. The transmission capacity, and therefore the infectious power of the aerosols, also depends on the presence of airborne particulates. One feature of all particulates is that they can convey and release toxic molecules, as well as microorganisms, including viruses [23]. Some studies have associated the spread of COVID-19 cases with PM2.5 microparticle pollution levels [24,25,26]. In Italy and other European countries, technical guidelines have been released to limit the operation of HVAC systems, such as the recirculation of exhausted air [27,28,29]. The different methods of air distribution have different limitations and solutions [30]. All safety measures, including those on the use of air-conditioning, have costs, which must be balanced by the benefits. The limitation of the use of air conditioners could create thermal discomfort, and negatively affect productivity and health outcomes in the workplace—where people spend one-third of their lifetime—and other indoor environments. The assessment of the risk of infection associated with the use of air-conditioning is essential for making correct decisions. Therefore, to evaluate the COVID-19 risk associated with the presence of air-conditioning systems, we conducted a rapid review of the literature concerning outbreaks of coronaviruses (SARS-CoV-1, MERS-CoV, and SARS-CoV-2) in indoor environments.

## 2. Materials and Methods

Due to time constraints related to the urgency of the ongoing pandemic, we carried out a rapid review [31], thus streamlining the rules that characterize a systematic review. We utilized the participants–exposure–comparisons–outcome (PECOS) criteria, and we defined them according to evidence-based practice [32]—P (participants) is human subjects residing in indoor environments, E (exposure) is exposed to air-conditioning systems (HVAC), C (comparisons) is any comparison between the pathogens under study, and O (outcome) is respiratory infection outbreaks caused by SARS-CoV-1, MERS-CoV, or SARS CoV-2. Only original studies (both observational and experimental researches) written in English were retrieved, with no limit on time. Narrative reviews, opinions, and commentaries were excluded. We also excluded experimental studies on the airborne transmission of coronaviruses that were not associated with outbreaks. Articles indexed in PubMed/MEDLINE, PubMed Central (PMC), Google Scholar databases, and medRxiv (a pre-print server for health sciences), were retrieved on July 11, 2020. A snowball strategy was then adopted to extend the search, including extensive cross-referencing, and using subject headings or keywords to search for similar relevant articles. Keyword checking was performed by the authors independently. The electronic search strategy used keywords related to the topic under investigation (“air-conditioning” OR “HVAC”) in combination with “COVID-19,” “virus,” “SARS,” “MERS,” “SARS-CoV-2,” and “coronavirus,” properly combined by Boolean operators. After independently reviewing all titles/abstracts to identify potentially relevant articles, an author (FC) selected studies for full-text review [32]. Data concerning the country of study, the study design, and the study setting, as well as the study’s outcomes, were extracted. The results of the studies retained in the present rapid review were synthesized qualitatively.

## 3. Results

In our review, we found 14 studies reporting outbreaks of coronavirus infection in indoor environments (Table 1).

Seven studies concerned SARS-CoV-1, and six SARS-CoV-2, respectively, while only one regarded MERS-CoV. Seven studies were carried out in Hong Kong, one in South Korea, three in Japan, and three in China. The studies were conceived as cross-sectional, in two cases with a retrospective collection of data, and in six cases complemented by an airflow modelling method or environmental sampling. In the SARS-CoV-1 epidemic, studies focused mainly on two outbreaks, which occurred in the Prince of Wales Hospital, a regional and teaching government hospital, and in Amoy Gardens, a private housing estate in Hong Kong, which were the most seriously affected locations during the 2003 outbreak of Severe Acute Respiratory Syndrome (SARS). Both were served by air-conditioning systems. The episodes occurred in 2003. In the first one, in March 2003, 156 patients in the Prince of Wales Hospital in Hong Kong were infected by a patient treated with a jet nebulizer [33]. In this episode, which affected healthcare workers, medical students, and patients, the proximity of the patients may have allowed the infection to spread through nuclei droplets, although the production of aerosols during treatment was evident.

This outbreak was also studied by Wong et al. [34], with a retrospective observational study on 66 medical students who visited the index patient’s ward. The authors showed that the flow rate was highest in the air supply diffuser in the index patient’s cubicle, and lowest in the corresponding exhaust grille. This imbalance would have favored the spread of aerosols. The computed concentration contours of aerosols, in fact, matched epidemiologic data. A retrospective study of on outbreak involving 74 patients in the same hospital indicated that the rapid evaporation of the droplets produced by coughing in a relatively dry, air-conditioned environment, could also induce virus-laden aerosol, which was probably responsible for spreading the infection to patients who were not in the same room [35]. The ward, however, was air-conditioned by a fan coil system with a separate fresh air supply. The exhaust air from this ward was discharged to the outside and not recirculated to other wards.

A modelling study conducted by Chen et al. [36] on Ward 8A of the same hospital in Hong Kong, where patients and staff were infected, demonstrated the direct responsibility of HVAC systems in the diffusion of the disease. The air exchange owing to temperature difference played a significant role in SARS transmission during the nosocomial outbreak in Ward 8A, and the two-way airflow effect at the openings could have played an important role in bioaerosol transmission [36]. The SARS outbreak that occurred in 2003 in the isolation ward of an infectious disease hospital in Hong Kong also had a spatial infection pattern compatible with airborne transmission. Some design defects in the air-distribution system, with unbalanced flow rates in supply diffusers and exhaust grilles, could have played a role in infection spreading. Computational fluid dynamics simulations confirmed this hypothesis [37]. In the same year, more than 300 residents of a private high-rise housing estate were infected with SARS CoV-1 within a short period, and 42 died. The case index was a tenant receiving haemodialysis. Virus-containing aerosol could have escaped into the narrow lightwell between the buildings and spread in rising air currents. The association between the predicted bioaerosol concentration and the spatial infection pattern suggested an airborne transmission route, and the aerosol distribution patterns coincided with the spatial and temporal distribution of infections [38,39]. Unfortunately, none of the investigations were completed with the isolation of microorganisms in the environment. In 2015, the spread of MERS was associated with aerosol transmission in three patients from two hospitals in South Korea [40]. Environmental sampling from the air exhaust damper revealed the presence of viral particles of MERS-CoV. The potential airborne transmission of MERS was supported by the disclosure of viral particles in the hospital environment including air, fomites, and environmental surfaces.

In summary, six of the seven SARS studies suspected that the air-conditioning system had played a role in the spread of the infection. In the only study available on MERS, contamination of HVAC by viral particles was demonstrated.

Data concerning the new coronavirus SARS-CoV-2 are obviously scarce. The analysis of 318 outbreaks involving, in different settings, 1245 infected individuals in 120 cities in China from 29 December to 11 February 2020, prompted Quian et al. to assert that long-range aerosol transmission had occurred in crowded spaces with poor ventilation. Detection of infectious particles in the air and on inaccessible surfaces suggested that the virus might be transmitted via airborne routes, and not only from droplet exposure [41]. Three studies were devoted to the outbreak that occurred in the Diamond Princess cruise ship, which affected 355 people in Japan. According to Zhang et al., the main route for transmission was from person to person, but other routes, including aerosol transmission via central air supply or drainage systems, could not be excluded [42]. The studies of Xu et al. [43] and Mizumoto and Chowell [44] on the same outbreak, led to the opposite conclusion. Most of the cases originated by passenger-to-passenger transmission through close contact and fomites. After the adoption of quarantine measures, new cases among passengers were limited to those who stayed in the same room with an infected passenger. Therefore, the ship’s central air-conditioning system would not have played a role, i.e., the long-range airborne route was not relevant in the outbreak. Another outbreak occurred in a Chinese restaurant from January 26 to February 10, 2020. Ten persons from three families were infected with SARS-CoV-2. The restaurant had an air-conditioned, fifth-floor building without windows. Each floor had its own air conditioner. According to Lu et al. [45], droplet transmission was likely prompted by air-conditioned ventilation. The key factor for the outbreak was the direction of the airflow. Studying the same outbreak with computer simulations, Li et al. [46] postulated that the infection distribution was consistent with a spread pattern representative of exhaled virus-laden aerosols.

In summary, in four out of the six studies on SARS-CoV-2, the diffusion of viral particles through HVAC was suspected or supported by computer simulation, while in the other two studies, it was excluded based on epidemiological considerations.

## 4. Discussion

The few COVID-19 investigations available do not provide sufficient evidence that HVAC systems can play an important role in the indoor outbreaks reported to date. Demonstration of the contamination of HVAC systems by SARS-CoV-2 viral particles is currently lacking. This evidence, in fact, was collected only during the MERS epidemic, and it is still unknown if the SARS-CoV-2 virus has the same potential transmission and spreading diffusion as the previous one. Furthermore, modelling studies on the distribution of viral particles for COVID-19, which in the first SARS epidemic strongly supported the possibility that SARS-CoV-1 aerosols could be captured and re-emitted by HVAC, are not yet available. Nevertheless, the findings obtained from the previous coronavirus epidemics, in which the HVAC systems were often held responsible for the spreading of the virus, do not allow us to believe that they are now totally risk-free. Furthermore, we know that certain medical procedures may produce aerosols, and that they have been responsible for the spread of coronavirus in healthcare settings [47]. Environmental investigations have shown the presence of SARS-CoV-2 viral particles on surfaces that cannot be reached by droplets, and this is indirect evidence that the transmission of aerosols has taken place [48,49]. Recent COVID-19 outbreaks reported during choir practice [50], in a call-center [51] and in fitness classes [52], have suggested the role of aerosol transmission in combination with droplet transmission, particularly in crowded and inadequately ventilated indoor spaces. Experimental studies in a laboratory-controlled environment demonstrated that aerosolized SARS-CoV-2 particles (<5 µm) remained suspended in the air for at least 3 h, viable in air for at least 1 h, and on surfaces for up to days [53,54,55]. The half-life of viral particles could be different in relation to meteorological conditions—such as temperature, relative humidity, and ultraviolet radiation—that could degrade and weaken the virus [56,57,58].

Ventilation systems have been already reported as a way of transmitting/spreading infectious diseases such as measles, chickenpox, flu, smallpox [59,60], and the 2009 influenza A (H1N1) pandemic [61]. The possibility that the SARS-CoV-2 virus remains suspended in the air, in free micro-drops or attached to particulates, and therefore can be picked up by the air-conditioning systems and put back into circulation, poses an evident safety problem for indoor environments [62,63]. The characteristics of HVAC systems, therefore, are very important for safety purposes. Unfortunately, the available studies refer exclusively to outbreaks that occurred in the Far East, in environmental and plant conditions that cannot be considered representative of other parts of the world. Building types, air-conditioning systems, regulations, and maintenance requirements may differ between countries, so the available studies may not be generalizable.

On the other hand, the accumulated knowledge about the novel SARS-CoV-2 indicates that it has higher aerosol and surface stability than SARS-COV-1 and can remain viable and infectious in aerosol for hours [64]. Patients affected by COVID-19 could have a high viral load, which correlates with transmission risk and disease severity [65]. When dealing with a new disease, particular caution is required.

The aerosol model of SARS-CoV-2 transmission has several practical implications, and this is confirmed by the analysis of the most important guidelines released by organizations and international agencies [27,28,29], which should be based on the best available scientific evidence. Most of the existing recommendations, which suggest deactivating air recirculation mode in all indoor environments, seem to be especially based on previous evidence of airborne transmission of SARS-CoV-1 and other airborne viruses, as research on SARS-CoV-2 is still limited. Only future studies will clarify whether HVAC implants played a role in favoring the spread of viruses in COVID-19. In the meanwhile, occupational preventive measures should be based on the best possible scientific evidence by balancing costs and effectiveness, yet according to the principles of a precautionary approach. Limiting recirculating air may not be technically possible in all workplaces, may give rise to additional costs, and could not balance cooling and heating functions in summer and winter, with appropriate levels of relative humidity and air velocity [66]. Moreover, too high, or too low temperatures, and low ambient humidity, are known to enhance viral transmission [67,68], and thus could harm the elderly and workers with “fragilities.” Therefore, employers, assisted by their consultants, must conduct a careful assessment of the risks and benefits associated with the use of air-conditioning systems. The choice to keep the systems in operation, guaranteeing comfortable working conditions, must always be accompanied by a continuous and scrupulous maintenance of the systems, with the use, where possible, of high-efficiency total filters. The technical and engineering characteristics of HVAC are of the utmost importance in the diffusion of pollutants. Undoubtedly the most effective methods to reduce pollution are the increase in ventilation (increase in air flow rates) and the minimization of air recirculation, with the introduction and treatment of external air. However, these methods are very expensive, and often not very efficient. All air intake systems should be designed so as to avoid the presence of air currents in the respiratory area of workers or occupants; this undoubtedly contributes to avoiding the long-distance transport of droplets and aerosols.

The efficiency of HVAC systems filters could be increased by nanomaterials [69]. In healthcare settings, environmental engineering controls, consisting of physical engineering elements such as negative pressure rooms, dilution ventilation, high-efficiency particulate air filtration, ultraviolet lights, and scavenging devices, can reduce the aerosol diffusion [70]. In other types of environment, proposed alternatives to HVAC include adopting natural ventilation and personalized ventilation–personalized exhaust systems (PV-PE) for microenvironments [61]. In the risk assessment process, it is of primary importance to estimate the probability that an infectious person is inside the premises. This probability can be inferred from the trend of the epidemic in the region where the building is located, and from the consideration that COVID-19 can spread in many people in an entirely asymptomatic way, making screening at the entrance ineffective [71]. It was observed that oligosymptomatic patients with a SARS-CoV-2 infection can have a greater viral load than that of patients hospitalized with severe forms [72], and therefore represent a real threat. Therefore, the risk assessment and the decision to keep the air-conditioning system active must be dynamic and based on the epidemiological evolution of the pandemic, as well as on the verification of the characteristics of the system and its efficiency.

This rapid review aimed to synthesize the available information for policy-makers, employers, technicians, and workers concerning the aerosol transmission of SARS, MERS and COVID-19 infections in air-conditioned indoor environments, in order to give a guide to the best choices for workers’ health protection. The weaknesses of our study are mainly in the data retrieved. Studies included in this review were few, often anecdotal, and published in non-peer-reviewed journals. The lack of quantitative data did not allow us to extract meta-analytical information. The “rapid” nature of this review, and finally the type of studies included, prevented us from making an appropriate evaluation of their quality. In conclusion, this short review is a warning for health and safety consultants, and a stimulus for further studies on the relationship between SARS-CoV-2 and air-conditioning systems.

## 5. Conclusions

The studies available to date are not sufficient to support the conclusion that air-conditioning systems favor the spread of the SARS-CoV-2 infection in office and indoor community environments. However, in previous coronavirus epidemics, HVAC systems not specifically designed to accommodate infectious patients were suspected of facilitating the spread of SARS-Cov-1 and MERS-CoV in hospital and community settings. The guidelines released by organizations and international agencies for controlling the spread of SARS-CoV-2 in indoor environments are based on these studies. The formation of aerosols, which is possible in many medical activities, and the great variability in the viral load in patients, make it necessary to adapt the safety of the HVAC systems to the specific control needs. Prevention in workplaces must be personalized, with the adoption of more stringent measures when epidemiological circumstances (e.g., increase in the number of cases, increase in hospital admissions) require it.

The precautionary principle requires the utmost attention in the design and management of air-conditioning systems, while waiting for further evidence on the effectiveness of safety measures proposed by international guidelines.

## Figures and Tables

**Table 1:** Studies on coronavirus outbreaks in indoor environments.

Authors and Year	Virus	Country	Type of Study	Type of Publication	Setting	Cases	Role of HVAC System
Lee et al., 2003 [33]	SARS-CoV-1	Hong Kong (SAR), China	Observational	Peer review journal	Hospital	156	Not considered (aerosol in the index case originated the outbreak)
Wong et al., 2004 [34]	SARS-CoV-1	Hong Kong (SAR), China	Retrospective	Peer review journal	Hospital	66	Indirectly proven by epidemiological data and ventilation study
Yu et al., 2005 [35]	SARS-CoV-1	Hong Kong (SAR), China	Retrospective	Peer review journal	Hospital	74	Indirectly proven by spatiotemporal pattern of infection
Chen et al., 2011 [36]	SARS-CoV-1	Hong Kong (SAR), China	Experimental modelling	Peer review journal	Hospital	Not reported	Indirectly proven by an airflow-dynamics model
Li, Huang et al., 2004 [37]	SARS-CoV-1	Hong Kong (SAR), China	Observational with experimental modeling	Peer review journal	Hospital	30	Indirectly proven by the spatiotemporal pattern of infection and by experimental modeling
Yu et al., 2004 [38]	SARS-CoV-1	Hong Kong (SAR), China	Observational with experimental modelling	Peer review journal	Community	187	Indirectly proven by the spatial and temporal pattern of infection and by an airflow-dynamics model
Li, Duan et al., 2005 [39]	SARS-CoV-1	Hong Kong (SAR), China	Experimental modeling	Peer review journal	Community	329	Indirectly proven by a multizone airflow-dynamics model
Kim et al., 2016 [40]	MERS	South Korea	Environmental sampling	Peer review journal	Hospitals	3	HVAC contamination was demonstrated
Quian et al., 2020 [41]	SARS-CoV-2	China	Observational	Pre-print	Community and workplace	1245	Suspected
Zhang et al., 2020 [42]	SARS-CoV-2	Japan	Observational	Peer review journal	Ship	355	Suspected
Xu et al., 2020 [43]	SARS-CoV-2	Japan	Observational	Pre-print	Ship	355	Not supported by the spatiotemporal distribution of cases
Mizumoto and Chowel, 2020 [44]	SARS-CoV-2	Japan	Observational	Peer review journal	Ship	355	Not supported by the spatiotemporal distribution of cases
Lu et al., 2020 [45]	SARS-CoV-2	China	Observational	Peer review journal	Restaurant	10	Suspected
Li, Qian, Hang, 2020 [46]	SARS-CoV-2	China	Observational with experimental modeling	Pre-print	Restaurant	10	Supported by computer simulation
